# Zinc-induced exposure of an LxL motif drives clathrin-mediated endocytosis of the zinc transceptor ZIP4

**DOI:** 10.1016/j.jbc.2026.111472

**Published:** 2026-04-17

**Authors:** Tianqi Wang, Chi Zhang, Yao Zhang, Majid Jafari, Kenneth M. Merz, Jian Hu

**Affiliations:** 1 Department of Biochemistry and Molecular Biology, Michigan State University, East Lansing, Michigan, USA; 2 Department of Chemistry, Michigan State University, East Lansing, Michigan, USA

**Keywords:** ZIP4, zinc, transporter, endocytosis, AP2, clathrin

## Abstract

ZIP4 functions as a zinc transceptor essential for dietary zinc absorption and undergoes zinc-dependent endocytosis to dynamically regulate zinc uptake capacity in response to changes in zinc availability. However, the detailed molecular mechanism of this posttranslational regulation remains elusive. In this work, we investigate the endocytosis-indispensable LxL motif to elucidate the molecular basis of ZIP4 endocytosis in HEK293T cells expressing ZIP4 exogenously and in cancer cell lines with endogenous ZIP4 expression. Integrating biochemical, cell and molecular biology, modeling, and computational approaches, our data collectively support a working model in which the LxL motif becomes more accessible at elevated cellular zinc levels and functions as a sorting signal that directly or indirectly associates with the adaptor protein complex 2 for clathrin-mediated endocytosis. These findings provide mechanistic insight into zinc-dependent ZIP4 endocytosis, advance our understanding of cellular zinc homeostasis, and establish a paradigm for studying substrate-induced endocytosis of other nutrient transceptors.

Some nutrient transporters act as transceptors, integrating substrate transport and substrate sensing to better maintain cellular nutrient homeostasis (1, 2, 3, 4). For many transceptors, the substrate-sensing function is manifested by substrate-induced endocytosis, in which a transceptor is removed from the plasma membrane when the level of substrate or substrate surrogate is elevated (2, 5, 6, 7, 8, 9, 10, 11, 12, 13, 14, 15, 16, 17, 18, 19), allowing cells to rapidly adjust transport capacity in response to changes in nutrient availability. Unravelling the molecular basis of this post-translational regulatory mechanism for transceptors is crucial for understanding nutrient homeostasis under physiological and pathological conditions (20, 21).

Among the known transceptors, ZIP4, a representative Zrt-/Irt-like protein (ZIP) metal transporter that is specifically expressed in the small intestine and the kidney for zinc uptake from food and zinc reabsorption from urine (21, 22, 23, 24, 25, 26), is a central player in maintaining systemic zinc homeostasis. At the cellular level, the zinc-sensing function of ZIP4 enables dynamic regulation of its cell surface expression, ensuring sufficient, but not excessive, zinc uptake for critical biological processes. ZIP4 undergoes internalization through two distinct mechanisms in response to changes in zinc availability. When zinc concentration rises significantly above physiological levels (tens to hundreds of micromolar), the second intracellular loop (hereafter the IL2 loop) of ZIP4 is ubiquitinated for subsequent degradation in both lysosome and proteasome (27). Zinc sensing in this process is conducted by the histidine-rich segment located in the IL2 loop (28). This long and highly variable loop has also been shown to be important for the noniron metal-induced endocytic degradation of a plant iron-transporting ZIP, iron-regulated transporter 1 (5, 29). In contrast, at physiological zinc concentrations (low micromolar range), ZIP4 undergoes constitutive, ubiquitination-independent endocytosis (referred to as zinc-dependent endocytosis) (21, 30). In our previous study, we showed that this constitutive endocytosis is significantly reduced upon zinc depletion by a combined treatment of N,N,N,N-tetrakis(2-pyridylmethyl) ethylenediamine (TPEN), a membrane permeable high-affinity zinc chelator and the culture medium treated with metal-chelating Chelex-100 resin, whereas transferrin internalization is unchanged under the same condition, indicating that the zinc-induced changes in endocytosis rate is specific for ZIP4 (31). By scanning potential zinc-binding sites, we showed that the transport site of human ZIP4 most likely functions as a zinc-sensing site (31). An extracellular zinc-binding site at the dimerization interface of mouse ZIP4 has also been suggested to play a role in zinc sensing (32). Although zinc binding to ZIP4 is proposed to induce conformational changes that enable its engagement with the endocytic machinery, the underlying biochemical and structural details remain unclear.

During membrane protein endocytosis, cargo recognition by adaptor protein is a critical step that involves specific protein–protein interactions. Various sorting signals have been identified for different endocytic mechanisms (33). In clathrin-mediated endocytosis, the heterotetrameric adaptor protein complex 2 (AP2), which is composed of α, β2, μ2, and σ2 subunits, plays a central role in cargo selection by either directly binding to cargo proteins *via* sorting motifs or by binding to cargo-specific adaptor proteins that bridge AP2 to the cargos (34, 35, 36). The best-characterized sorting motifs that are recognized by AP2 include the tyrosine-based motif (YxxØ, where Ø is a bulky hydrophobic amino acid) and the dileucine motif ([D/E]xxxL[L/I]) (33, 35), while many other sorting signals have been identified and more are expected to exist (35, 37). In our previous study on human ZIP4 endocytosis, we systematically scanned the IL2 loop and identified a conserved ^452^LQL^454^ segment essential for ZIP4 endocytosis (31). However, the exact role of this segment in zinc-dependent ZIP4 endocytosis remains unknown.

In this study, we employed multidisciplinary approaches to investigate the role and structural changes of the LQL segment in ZIP4 endocytosis. Using a flow cytometry-based antibody uptake assay, we demonstrated that the hydrophobicity of the two leucine residues is essential for ZIP4 internalization. Using a custom anti-ZIP4 mAb, we found that endogenously expressed ZIP4 in cancer cell lines undergoes a clathrin-mediated endocytosis. Importantly, our cell biology, biochemical, computational, and modeling data support a model in which both the LxL motif, functioning as an atypical dileucine sorting signal, and the dileucine motif-binding pocket in the AP2 σ2 subunit are critical for the association of ZIP4 with AP2. Live-cell cysteine accessibility assays performed on a series of single cysteine variants revealed that the IL2 loop undergoes significant zinc-dependent conformational changes. Together, these findings provide mechanistic insights into the molecular basis of zinc-dependent ZIP4 endocytosis.

## Results

### Essential roles of the leucine residues in the LQL segment

**Figure 1:**
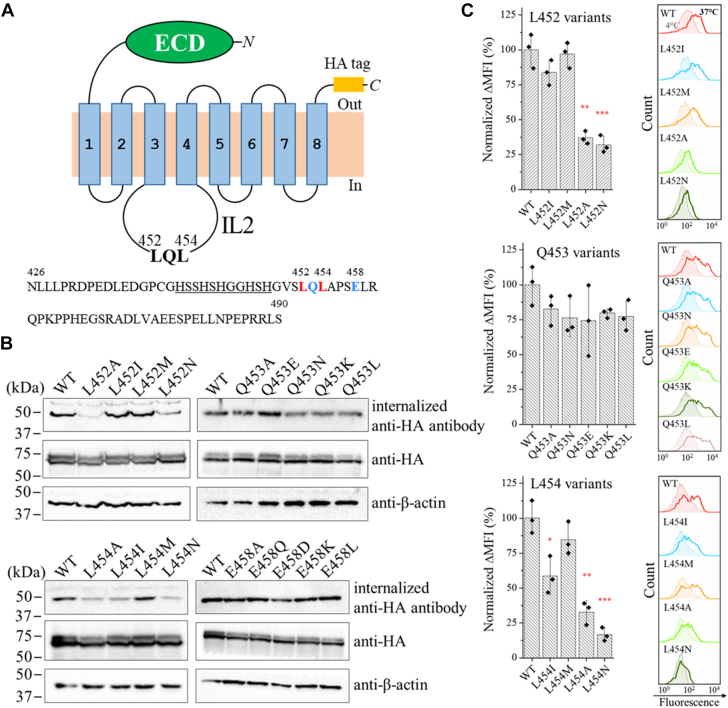
**Effects of the LQL mutations on human ZIP4 endocytosis in HEK293T cells.***A,* topology of hZIP4-HA in the plasma membrane. The LQL segment is located in the IL2 loop, whose sequence is shown and the residues studied in (*B*) are highlighted in *red* (indispensable) or *blue* (dispensable). The His-rich segment is *underlined*. The ECD is shown as a *green oval*. The HA tag (*orange rectangle*) is exposed to the extracellular space. *B,* effects of substitutions of the residues in the LQL segment on human ZIP4-HA endocytosis. E458 is a generally conserved residue in ZIP4 and thus was tested along with the LQL sequence. The internalized mouse anti-HA antibodies were detected by Western blot using an horseradish peroxidase-conjugated anti-mouse antibody. The total expression levels of ZIP4 variants were detected by an anti-HA antibody. Western blot of anti-β actin was used as loading control. *C,* comparison of anti-HA antibody uptake between WT ZIP4-HA and its variants using a flow cytometry–based assay. ΔMFI was calculated by subtracting the MFI of the cells incubated with the antibody at 4 °C from the MFI of the cells incubated with the antibody at 37 °C. The values of mean ΔMFI were then normalized by setting the value for WT ZIP4-HA as 100%. Each column chart shows the data in one of three independent experiments with three biological replicates (shown as *solid diamond*) tested for each condition. The error bars indicate 1 ± S.D. The representative histograms on the *right* show the profiles of 4 °C (*shaded*) and 37 °C (*open*). Statistical analyses were performed using two-tailed Student’s *t* test. The *p* values are 0.0011 (L452A) 0.00099 (L452N), 0.015 (L454I), 0.0012 (L454A), and 0.00033 (L454N), respectively. ∗*p* < 0.05; ∗∗*p* < 0.01; and ∗∗∗*p* < 0.001. ZIP, Zrt-/Irt-like protein; MFI, mean fluorescence intensity; ΔMFI, difference of MFI; ECD, extracellular domain; HA, hemagglutinin; HEK, Human embryonic kidney.

Our previous study has shown that alanine substitution of the residues in the LQL segment diminished ZIP4 endocytosis (31). In this work, we conducted an extensive mutagenesis study on these residues to determine what properties of the amino acids at these positions are important. Antibody uptake/feeding assay has been widely used in the study of endocytosis of ZIP4 and other membrane proteins (30, 32, 38, 39). To do so, the C-terminal hemagglutinin (HA)-tagged human ZIP4 and its variants were transiently expressed in Human embryonic kidney (HEK293T) cells (Fig. 1*A*). After incubation with the anti-HA antibody at 37 °C to allow antibody uptake, cell lysates were applied to Western blot to detect the internalized antibody (Fig. 1*B*). For the first leucine (L452), replacement with either isoleucine or methionine did not affect antibody uptake, but alanine or asparagine substitution nearly abolished antibody uptake. Similarly, alanine or asparagine substitution of the second leucine (L454) led to largely reduced antibody uptake. Although the substitution with methionine did not affect endocytosis, the substitution by isoleucine significantly reduced it. In contrast, none of the substitutions on the glutamine residue (Q453) reduced antibody uptake. We also tested substitutions on E457, a generally conserved residue close to the LQL segment, and the results indicated that amino acid replacement at this position had no effect on ZIP4 endocytosis.

Given that the Western blot–based assay barely allows a statistical analysis, we applied a flow cytometry–based antibody uptake assay to study ZIP4 endocytosis (Fig. S1). In this assay, cells transiently expressing ZIP4 or its variants were incubated with a fluorescence-labeled anti-HA antibody at 4 °C or 37 °C for 45 min and then washed with an acidic buffer at 4 °C to remove the cell surface bound antibody before flow cytometry analysis. The differences in mean fluorescence intensity (MFI) between the samples treated at 4 °C and 37 °C represent the signals derived from the internalized antibodies. Consistent with the results obtained from the Western blot experiments, antibody uptake was significantly reduced for the L452A, L452N, L454A, and L454N variants (Fig. 1*C*), indicating that the hydrophobicity of L452 and L454 is important for ZIP4 endocytosis. Substitution of L454 with isoleucine significantly reduced endocytosis, whereas substitution with methionine had little effect. As the results of the Q453 variants showed that changes in charge, size, and polarity at this position did not affect ZIP4 endocytosis, the LQL segment is henceforth referred to as the LxL motif.

### Development of an anti-ZIP4 mAb to recognize endogenously expressed ZIP4

Chlorpromazine, an endocytosis inhibitor believed to disrupt the formation of clathrin-coated pits (40), was shown to dose dependently reduce ZIP4 endocytosis in HEK293T cells (31), suggestive of a clathrin-mediated endocytosis. Given that the endocytosis mechanism for overexpressed proteins may differ from that for endogenously expressed proteins, we developed an anti-human ZIP4 mAb (Clone 1G3B2) to study ZIP4 endocytosis in HepG2 cells, a human hepatocyte cancer cell line in which ZIP4 mRNA and protein expression have been documented in Human Protein Atlas https://www.proteinatlas.org/ENSG00000147804-SLC39A4/cell+line and ProteomicsDB https://www.proteomicsdb.org/protein/67007/expression, respectively. The mouse-derived 1G3B2 mAb, generated using the purified extracellular domain (ECD) of human ZIP4 as antigen (Fig. S2*A*), stained the live HEK293T cells overexpressing ZIP4, whereas the empty vector-transfected cells showed no staining in immunofluorescence imaging (Fig. S2*B*), indicating that the 1G3B2 mAb specifically recognizes human ZIP4 in its native state.

### Clathrin-mediated and AP2-dependent ZIP4 endocytosis

**Figure 2:**
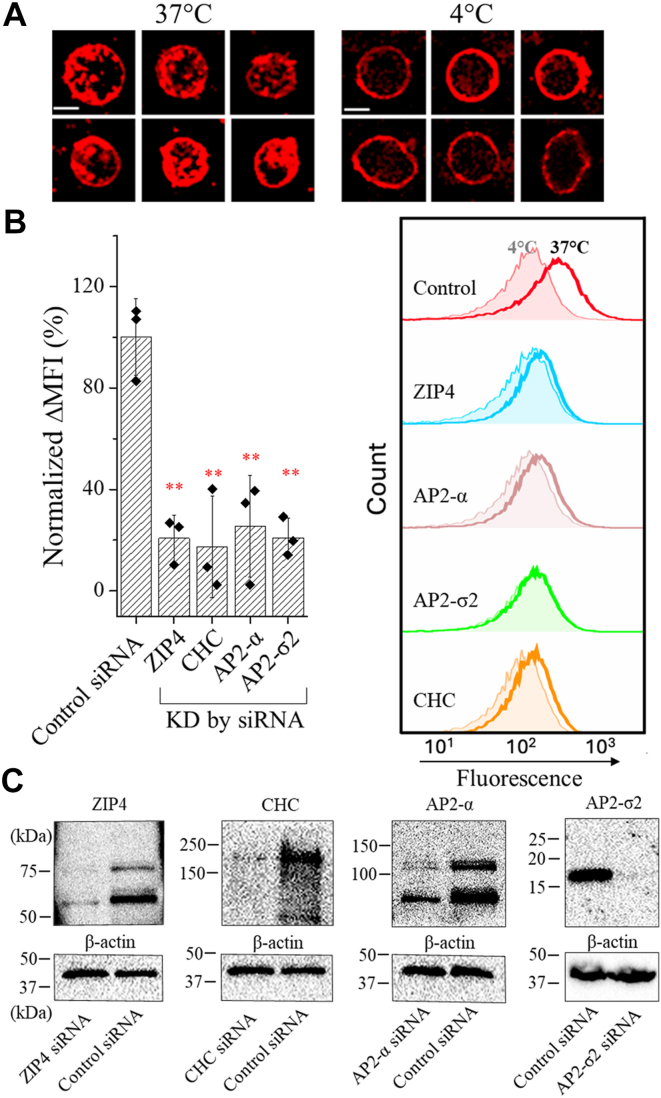
**Endocytosis of endogenously expressed ZIP4 in HepG2 cells.***A,* internalization of the 1G3B2 anti-ZIP4 mAb by HepG2 cells. Live HepG2 cells in suspension were incubated with 1G3B2 mAb at 4 °C and 37 °C, respectively. After wash, fixation, and permeabilization, cells were stained with an Alexa Fluor 568–conjugated anti-mouse antibody. Immunofluorescence images were taken using a confocal microscopy. Representative cells from multiple images were shown. Scale bars represent 10 μm. *B,* analysis of 1G3B2 mAb uptake by flow cytometry. The target proteins were knocked down by siRNA. ΔMFI was calculated by subtracting the MFI of the cells incubated with the antibody at 4 °C from the MFI of the cells incubated with the antibody at 37 °C. The values of mean ΔMFI were then normalized by setting the value of the control group as 100%. The column chart shows the data in one of two independent experiments with three biological replicates (shown as solid diamond) tested for each condition. The error bars indicate 1 ± S.D. The representative results are shown in histogram chart on the right. Statistical analyses were conducted using two-tailed Student’s *t* test. The *p* values are 0.0068 (ZIP4), 0.0013 (clathrin heavy chain, CHC), 0.0047 (AP2-α), and 0.0017 (AP2-σ2), respectively. ∗∗*p* < 0.01. *C*, knockdown of the target proteins by control siRNA or specific siRNA. The expression levels were analyzed in Western blot using the corresponding antibodies. ZIP, Zrt-/Irt-like protein; MFI, mean fluorescence intensity; ΔMFI, difference of MFI; ECD, extracellular domain; HA, hemagglutinin; HEK, Human embryonic kidney; AP2, adaptor protein complex 2.

We then used the 1G3B2 mAb to stain the live HepG2 cells and the result confirmed the expression of ZIP4 in this cell line and also showed that the antibody was internalized when cells were incubated at 37 °C, but barely at 4 °C (Fig. 2*A*). Using a similar flow cytometry–based antibody uptake assay, the MFI difference between the cells incubated at 37 °C and 4 °C was found to increase over time (Fig. S2*C*). To confirm the antibody uptake is ZIP4-specific, ZIP4 was knocked down by siRNA and the greatly diminished antibody uptake indicated that the 1G3B2 mAb can be used as a tracer to study ZIP4 endocytosis in HepG2 cells (Fig. 2*B*). Although no obvious difference in MFI between control and ZIP4-knockdown cells at 4 °C was detected by flow cytometry under the current experimental conditions, immunofluorescence staining (Fig. S2*D*) clearly demonstrates that cell surface ZIP4 expression is markedly reduced in ZIP4-knockdown cells.

To test the possible involvement of clathrin in ZIP4 endocytosis in HepG2 cells, as suggested by the inhibition of ZIP4 endocytosis in HEK293T cells by chlorpromazine (31), we knocked down clathrin heavy chain using siRNA and found that the 1G3B2 mAb uptake was largely abolished (Fig. 2*B*), indicating that ZIP4 endocytosis is a clathrin-mediated process. Since AP2 is a central player in clathrin-mediated endocytosis, we tested whether it is required for ZIP4 endocytosis by individually knocking down the α and σ subunits using siRNAs. The flow cytometry data showed drastically reduced antibody uptake (Fig. 2*B*), indicating that AP2 is essential for ZIP4 endocytosis.

**Figure 3:**
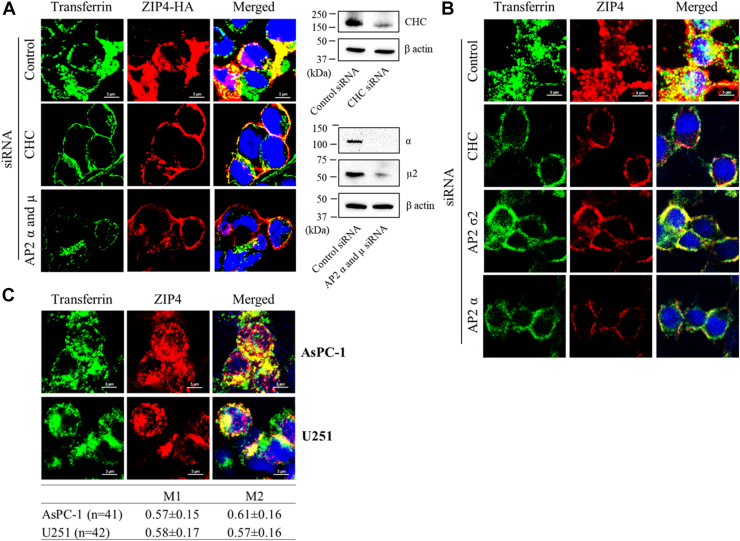
**Cellular uptake of transferrin and ZIP4-detecting antibodies visualized by confocal microscopy.***A,* comparison of the uptake of Alexa 488–conjugated transferrin and Alexa Fluor 647–conjugated anti-HA antibody by HEK293T cells transiently expressing a C-terminal HA-tagged human ZIP4. Knockdown of clathrin heavy chain (CHC), AP2-α, and AP2-μ were examined by Western blot using the corresponding antibodies. *B,* comparison of the uptake of Alexa 488–conjugated transferrin and anti-ZIP4 1G3B2 mAb by HepG2 cells that express ZIP4 endogenously under various knockdown conditions. *C,* uptake of Alexa 488–conjugated transferrin and anti-ZIP4 1G3B2 mAb by human pancreatic adenocarcinoma cell line AsPC-1 and human glioblastoma multiforme cell line U251 that express ZIP4 endogenously. Manders’ colocalization coefficients (MCCs) were calculated on cells (n, cell number) from two independent experiments using ImageJ, and the M1 and M2 values (mean ± SD) are shown in the table. In (*B* and *C*), the anti-ZIP4 antibodies were detected using an Alexa Fluor 568–conjugated goat anti-mouse IgG. Scale bars represent 5 μm. ZIP, Zrt-/Irt-like protein; HA, hemagglutinin; AP2, adaptor protein complex 2.

To further confirm that ZIP4 endocytosis is a clathrin-mediated and AP2-dependent process, we used confocal microscopy to compare the cellular localization of ZIP4 with that of transferrin. The transferrin receptor is a well-characterized cargo of clathrin-mediated endocytosis and is recognized by the μ2 subunit of AP2. As shown in Figure 3*A*, HEK293T cells transfected with control siRNA efficiently internalized both fluorescence-labeled transferrin (in all cells) and the anti-HA antibody (in cells transiently expressing the HA-tagged ZIP4). Two proteins colocalized in intracellular punctate structures, indicating that they are within the same endocytic vesicles/endosomes. Knockdown of clathrin heavy chain by siRNA markedly abolished the uptake of both transferrin and anti-HA antibody, resulting in their retention at the plasma membrane. Same results were obtained when the AP2 subunits α and μ2 were simultaneously depleted. In HepG2 cells where ZIP4 is endogenously expressed, colocalization of transferrin and the 1G3B2 anti-ZIP4 mAb was observed in the intracellular puncta and the uptake of either protein was abolished by the knockdown of clathrin heavy chain, AP2 α or σ2 subunit (Fig. 3*B*). Colocalization of transferrin and the 1G3B2 mAb were also observed in two additional human cancer cell lines known for ZIP4 expression, pancreatic adenocarcinoma AsPC-1 (41), and glioblastoma multiforme U251 (42) (Fig. 3*C*). These findings strongly indicate that endocytosis of endogenous ZIP4 in HepG2 cells proceeds *via* the same clathrin-mediated, AP2-dependent mechanism as observed for exogenously expressed ZIP4 in HEK293T cells. Consistently, the measured Manders’ colocalization coefficients for AsPC-1 and U251 cells indicated colocalization of a substantial fraction of transferrin and the anti-ZIP4 mAb (Fig. 3*C*).

Given that AP2 may directly bind to the cargo protein or engage indirectly by binding to a cargo-specific adaptor protein that recognizes the cargo protein (35), these results raised a question of the exact role of AP2 in ZIP4 endocytosis.

### Predicted structural model of the AP2–IL2 complex

**Figure 4:**
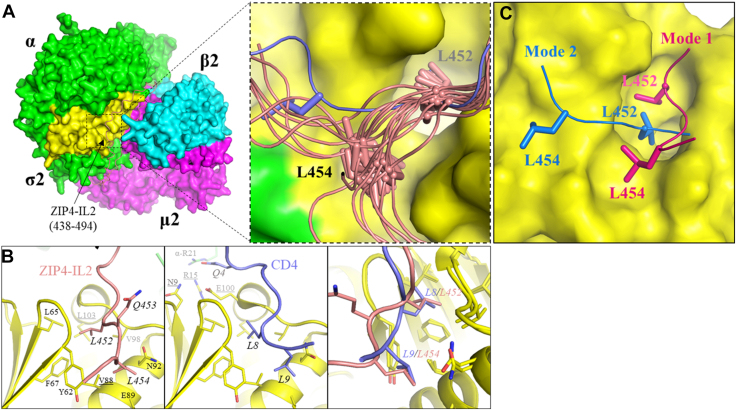
**Structural model of the AP2–IL2 complex generated by AlphaFold 3.***A,* overall structure (*left*) and zoomed-in view (*right*). AP2 is shown in surface mode and the ZIP4-IL2 peptide (438–494, *pink*) is in *cartoon mode* (*left*). Sixteen of thirty-five predicted models showed a consistent binding of the LxL motif to a hydrophobic pocket in the σ2 subunit (*right*). The model in *blue* is different from the other models and was shown to be an alternative binding mode in MD simulations. L452 and L454 are depicted in *stick mode*. *B,* comparison of the binding of the LxL motif (*left*) and the canonical dileucine motif in CD4 (*middle,* PDB: 2JKR). The superimposed structures are shown in the *right panel*. The residues mutated in this work (V88, L103, N9, R15, and E100 in the σ2 subunit) are *underlined*. *C,* two binding modes revealed by MD simulations of the predicted σ2–IL2 complex. For clarity, only five residues in the LxL-containing peptide are shown. AP2, adaptor protein complex 2; IL2, second intracellular loop; MD, molecular dynamics; ZIP, Zrt-/Irt-like protein.

To look for a potential binding between ZIP4 and AP2, we used AlphaFold 3 (43) to generate the structural models of the AP2-IL2 complex using the intact AP2 and a peptide corresponding to the IL2 loop of human ZIP4 as input (Fig. 4*A*). The generated AP2 structure models showed a consistent open-like conformation when compared to the experimentally solved open state AP2 complex structures (Fig. S3) (44, 45, 46). Remarkably, in 28 of 35 models, the IL2 loop peptide was predicted to bind to a hydrophobic pocket in the σ2 subunit that is known for the binding of the canonical dileucine motif (47). Of the 28 models, the sequence of ^477^ESPELL^482^, which is consistent with the canonical dileucine motif, was found to bind to the hydrophobic pocket in three cases (Fig. S4*A*). However, our previous mutagenesis study has shown that alanine substitutions of the leucine residues in this segment had no effect on ZIP4 endocytosis (31). Nine models showed the binding of a segment containing ^472^LV to the same pocket (Fig. S4*B*), but this segment was also ruled out in the previous screening. Of great interest, 16 models showed the binding of the LxL motif to this hydrophobic pocket (Fig. 4*A*). Structural comparison revealed a similar binding mode for the canonical dileucine motif (a CD4-derived peptide, PDB IDs: 2JKR and 6QH6) (47, 48) and the LxL motif (Fig. 4*B*). The first leucine residue (L8 in the CD4 peptide and L452 in ZIP4) occupies the same hydrophobic position deep in the pocket; in contrast, the second leucine residue (L9 in CD4 and L454 in ZIP4) binds to a shallower and more polar position that is surrounded by Y62, E89, and N92 and the isopropyl groups on their side chains are superimposable. Structural differences are also remarkable. The two consecutive leucine residues in CD4 form a turn in order to position their side chains on the same side of the backbone. For the LxL motif, because a residue is inserted between the two leucine residues, the backbone of the LxL motif adopts a β-strand–like conformation, positioning the side chains of the two leucine residues on the same side of the backbone for the binding to the hydrophobic pocket. As a result, the x residue points away from the binding pocket and is not involved in the binding to the σ2 subunit.

To assess the stability of the predicted interaction between the LxL motif and the hydrophobic pocket of the σ2 subunit, we performed molecular dynamics (MD) simulations. In 500 ns simulations, the peptide remained associated with the σ2 subunit while two binding modes emerged. In one mode, both leucine residues in the LxL motif stay at the positions as predicted. In the other mode, the second leucine (L454) moved from the initial position to a nearby shallow cavity (Fig. 4*C*), and this alternative binding mode was predicted by AlphaFold 3 as a minor species (blue peptide in Fig. 4*A*).

### Essential role of the dileucine motif-binding pocket in ZIP4 endocytosis

**Figure 5:**
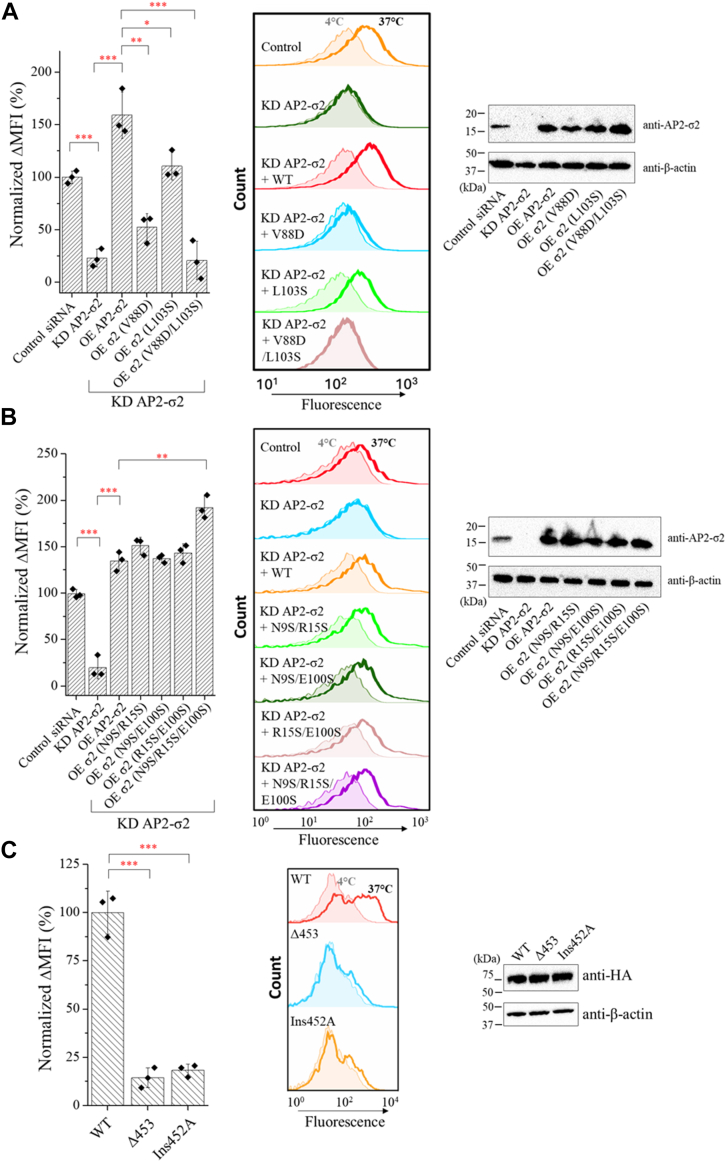
**Identification of the key residues in the AP2–IL2 interaction.***A,* the importance of the hydrophobic pocket in the σ2 subunit. The σ2 subunit in HepG2 cells was knocked down by siRNA and rescued by overexpressing an siRNA-resistant σ2 subunit or its variants with hydrophobic residues (V88 and L103) substituted with polar/charged amino acids. ΔMFI was calculated by subtracting the MFI of the cells incubated with the antibody at 4 °C from the MFI of the cells incubated with the antibody at 37 °C. The values of mean ΔMFI were then normalized by setting the value of the control group as 100%. The column chart shows the data in one of two independent experiments with three biological replicates (shown as *solid diamond*) tested for each condition. The error bars indicate 1 ± SD. The representative results are shown in histogram chart (*middle*). Statistical analyses were conducted using two-tailed Student’s *t* test. The *p* values from *left* to *right* are 0.00020, 0.00056, 0.0020, 0.031, and 0.0011, respectively. ∗: *p* < 0.05; ∗∗: *p* < 0.01; and ∗∗∗: *p* < 0.001. The expression levels of the WT σ2 subunit or its variants were examined by Western blot, and β-actin was used as loading control. *B,* examination of the involvement of the electrostatic patch (composed of N9, R15, and E100) in the σ2 subunit. The knockdown and rescue experiments and Western blot were conducted in the same as in (*A*). The *p* values from *left* to *right* are 0.00043, 0.00027, and 0.0035, respectively. *C,* endocytosis of WT ZIP4-HA and its variants with changed distance between the two leucine residues in the LxL motif. The constructs were transiently expressed in HEK293T cells and the anti-HA antibody uptake assay was conducted and detected as described in Figure 1*C*. For (*A*–*C*), the column chart (*left*) shows the data in one of two independent experiments with three biological replicates tested for each condition. AP2, adaptor protein complex 2; IL2, second intracellular loop; ZIP, Zrt-/Irt-like protein; MFI, mean fluorescence intensity; ΔMFI, difference of MFI; HA, hemagglutinin; HEK, Human embryonic kidney.

To confirm the importance of the σ2 subunit in ZIP4 endocytosis, HepG2 cells were cotransfected with the siRNAs, which knocked down the endogenous σ2 subunit, and a vector harboring the gene encoding an siRNA-resistant WT σ2 protein. The results showed that overexpression of the WT σ2 protein not only rescued the attenuated ZIP4 endocytosis but also increased the endocytosis level higher than that in the cells transfected with control siRNA (Fig. 5*A*). Since the hydrophobicity of the pocket in the σ2 subunit has been shown to be important for the binding of canonical dileucine motif, we applied two functionally compromised variants (V88D and L103S) to the rescue experiment (47). Compared to the cells overexpressing the WT σ2 protein, the cells expressing either of these variants showed a significantly reduced rescue effect, and when the two mutations were combined (V88D/L103S), the rescue effect was completely eliminated (Fig. 5*A*). These results indicated an essential role of the dileucine motif-binding pocket in ZIP4 endocytosis.

Comparison of the binding modes for the canonical dileucine motif and the LxL motif (Fig. 4*B*) revealed a key difference: unlike the canonical motif, the LxL motif does not involve an acidic residue that interacts with the polar residue cluster (N9, R15, and E100) in σ2 through electrostatic interactions. To experimentally examine the role of this polar cluster in ZIP4 endocytosis, we tested additional σ2 variants in which the polar residues that form the electrostatic interactions with the acidic residue (or a glutamine in the case of CD4) in the canonical dileucine motif were replaced with serine residues (Fig. 5*B*). Previous studies have shown that disruption of this interface reduces the binding affinity for CD4-derived peptide (47), but our rescue experiments using these variants showed that none of these mutations compromised the rescue efficacy, suggesting that the binding of the LxL motif to the hydrophobic pocket of the σ2 subunit does not involve electrostatic interactions. Accordingly, this result substantially excluded the possibility that AP2 binds to a yet identified ZIP4-specific adaptor that uses a canonical dileucine motif for AP2 engagement. Taken together, the knockdown/rescue and mutagenesis experiments indicated that the hydrophobic pocket known for the binding of canonical dileucine motif in σ2 is essential for ZIP4 endocytosis, while the polar cluster used to stabilize dileucine motif does not play a role.

Using the same approach, we also tested that if a change in the distance between the two leucine residues in the LxL motif could be tolerated. To test this, we generated two ZIP4-HA variants, Δ453, in which Q453 was deleted, and Ins452A, in which an alanine was inserted after L452, and expressed them in HEK293T cells for the flow cytometry–based antibody uptake assay. As shown in Figure 5*C*, expression of either variant did not lead to efficiently internalized, indicating that an LL sequence (in the Δ453 variant) or an LAQL sequence (in the Ins452A variant) is unable to support ZIP4 endocytosis. The lack of an acidic residue upstream of the LL sequence may explain why the Δ453 variant failed to engage σ2 as the canonical dileucine motif. The increased spacing between the two leucine residues in the Ins452A variant likely disrupted the binding to a small hydrophobic pocket. This result suggests that the LxL motif likely binds to a pocket with limited volume and flexibility.

### Pulldown of σ2 and AP2 by ZIP4

**Figure 6:**
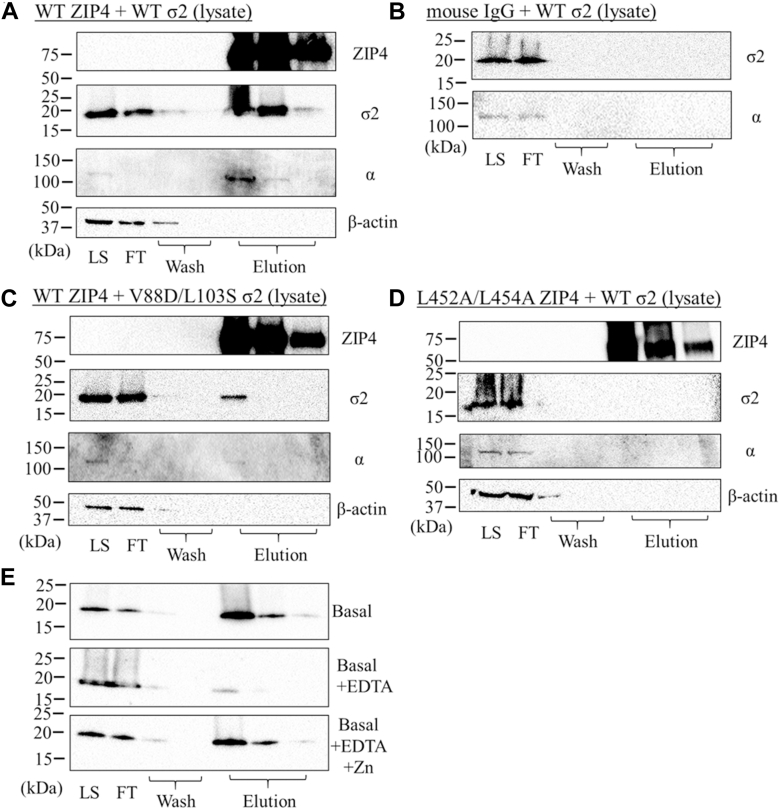
**Examination of ZIP4-AP2 association by pulldown.** The purified N-terminal FLAG-tagged ZIP4 was immobilized to protein G resin *via* the M2 anti-FLAG antibody. *A,* pulldown of the overexpressed WT σ2 and endogenous α subunits of AP2 in the lysates of HepG2 cells by WT ZIP4. *B,* control sample for (*A*), in which an irrelevant mouse IgG was immobilized to protein G resin. *C,* pulldown of the overexpressed V88D/L103S variant of σ2 and endogenous α subunits of AP2 in the lysates of HepG2 cells by WT ZIP4. *D,* pulldown of the overexpressed WT σ2 and endogenous α subunits of AP2 in the lysates of HepG2 cells by the AQA variant of ZIP4 (L452A/L454A). *E,* pulldown of the overexpressed WT σ2 in the lysates of HepG2 cells by WT ZIP4 in the presence of 1 mM EDTA (*middle*) or 1 mM EDTA plus 1.5 mM ZnCl_2_ (*bottom*) in comparison with the untreated sample (*top*). The volume of cell lysate is approximately ten times larger than the volume of each elution. In Western blots, ZIP4, σ2 subunit, α subunit, and β-actin were detected by the M2 anti-FLAG antibody, anti-σ2 subunit antibody, anti-α subunit antibody, and anti-β-actin antibody, respectively. The data shown are from one of at least two independent experiments that yielded similar results. LS, lysate; FT, flow through; AP2, adaptor protein complex 2; ZIP, Zrt-/Irt-like protein.

Since the hydrophobic pocket in σ2 is a top candidate for the binding of the LxL motif, we examined whether ZIP4 can physically associate with σ2 in cell lysate using a pull-down assay. To use ZIP4 as bait, the purified N-terminal FLAG-tagged human ZIP4 was bound to a Protein G resin *via* the M2 anti-FLAG antibody. The ZIP4-loaded resin was then used to capture binding partners from the lysates of HEK293T cells overexpressing an untagged σ2 (Fig. 6*A*). The σ2 subunit was found to coelute with ZIP4 upon elution with a low-pH buffer, whereas no σ2 was detected when the M2 antibody was replaced with an irrelevant mouse IgG in ZIP4 immobilization (Fig. 6*B*). This result strongly indicates a physical association between ZIP4 and σ2, either directly or indirectly. Notably, although only a portion of σ2 in lysate was bound to the ZIP4-loaded resin, nearly all of the endogenous α subunit was retained and coeluted with ZIP4. Replacement of the LQL sequence in ZIP4 with AQA (L452A/L454A, Fig. 6*C*) or introduction of V88D/L103S substitutions in σ2 (Fig. 6*D*) markedly reduced the pulldown of both σ2 and α subunits, suggesting that both the LxL motif of ZIP4 and the hydrophobic pocket of σ2 are essential not only for the association between ZIP4 and σ2 but also for the association of ZIP4 with the α subunit. Therefore, the α subunit must associate with ZIP4 indirectly *via* σ2, likely in the intact AP2 complex or the α-σ2 hemi complex. Overall, the pulldown results showed that ZIP4 and the σ2 and α subunits can form a complex, but whether their interactions are direct or indirect cannot be determined. Using the same pull-down assay, we also found that their association is zinc-dependent, as adding EDTA to the lysate drastically reduced the coelution and zinc supplementation restored it (Fig. 6*E*).

### Zinc-dependent conformational changes of the IL2 loop

**Figure 7:**
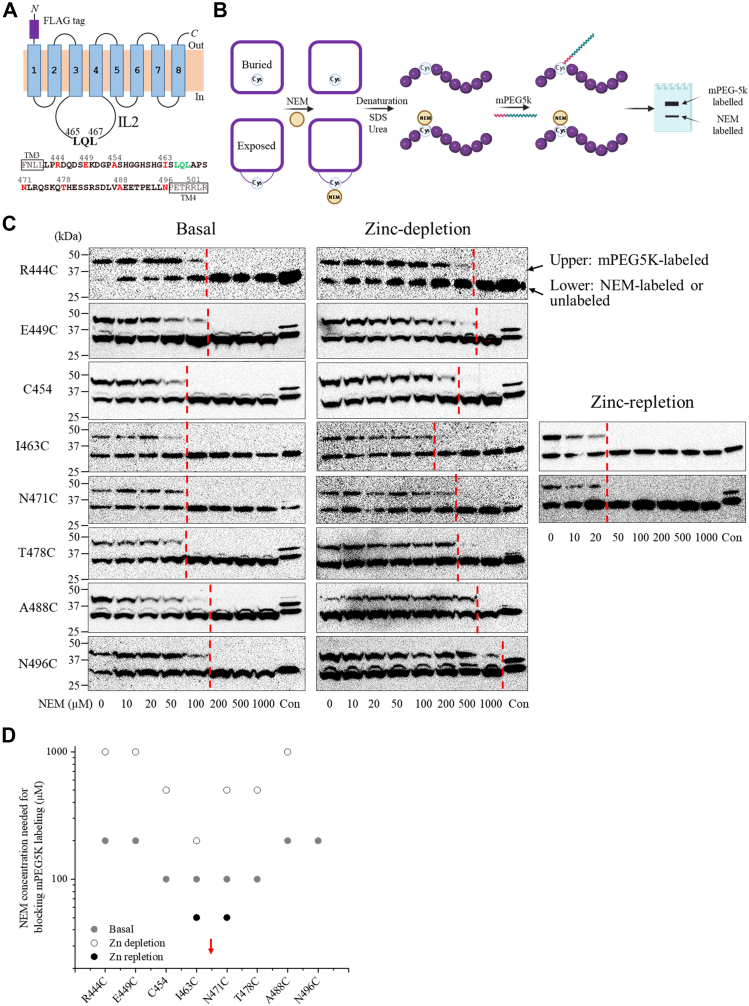
**Cysteine accessibility assay of mouse ZIP4 expressed in HEK293T cells.***A,* topology of the cysteine-free TMD of mouse ZIP4 (CysFree-TMD). The amino acid sequence of the IL2 loop is *bolded* and the residues substituted with cysteines are highlighted in *red*. The LxL motif is shown in *green*. Note that a cysteine residue is present at the position of 454 in WT ZIP4. *B, cartoon illustration* of the experimental procedure. *C,* cysteine accessibility assays of eight single-cysteine variants. For each variant, NEM titration was conducted and the *red dashed line* indicates the concentration of NEM above which no cysteine was labeled by mPEG5K. Cells in control group (Con) were not treated with NEM or mPEG5K. An anti-FLAG antibody was used in Western blot experiments. The shown data are the results of one of two independent experiments with similar results. *D,* analysis of NEM concentrations needed to completely block mPEG5K labeling for single-cysteine variants. Data were derived from the *red dashed lines* in (*C*). The *red arrow* indicates the position of the LxL motif. Even at the highest tested concentration of NEM, N496C under zinc depletion condition was still partially labeled with mPEG5K and therefore there is no data point for this variant. ZIP, Zrt-/Irt-like protein; IL2, second intracellular loop; NEM, N-ethylmaleimide.

To characterize zinc binding–induced conformational changes of IL2, which is an essential step in zinc-dependent endocytosis, we conducted a cysteine accessibility assay on mouse ZIP4 expressed in live HEK293T cells. Mouse ZIP4 was chosen is because it appears to be more stable than human ZIP4 in HEK293T cells according to the previous mutagenesis studies and therefore is likely to be more tolerant to mutations required for cysteine accessibility assay (21, 49). As there are 15 endogenous cysteine residues in the WT mouse ZIP4, including eight in the ECD forming four disulfide bonds as shown in the structure of ZIP4-ECD (50) and seven in the transmembrane domain (TMD), we removed all of cysteines by deleting the ECD, which is not required for ZIP4 endocytosis or zinc sensing (31, 32), and replacing the seven cysteine residues in the TMD with alanine, generating a variant named cysteine-free TMD (hereafter CysFree-TMD). Cell-based transport assay indicated that CysFree-TMD is a functional Zn^2+^ transporter and undergoes endocytosis as WT ZIP4 (Fig. S5). Then, cysteine residues were introduced to nonconserved positions in the IL2 loop of CysFree-TMD, resulting in eight single-cysteine variants (Fig. 7*A*).

The variants were transiently expressed in HEK293T cells and the accessibility of the introduced cysteine was studied on live cells under basal, zinc depletion, and zinc repletion conditions (Fig. 7*B*). Cells expressing N-terminal FLAG-tagged CysFree-TMD variants were first incubated with a membrane-permeable thiol-reacting reagent, N-ethylmaleimide (NEM), to label the exposed cysteines with NEM. After removal of residual NEM by extensive washing, cells were lysed in a denaturation/reducing solution to completely unfold proteins to expose buried cysteine residues for the subsequent reaction with excessive monofunctional PEG-maleimide 5K (mPEG5K), a thiol-reacting reagent with a molecular weight of 5 kDa. The resulting mixtures were then analyzed in Western blot to differentiate the exposed cysteine residues, which would show no band shift due to the low molecular weight of NEM, from the buried cysteine residues, which would show a 5 kDa increase in molecular weight due to the PEG conjugation. The ratio of the two bands reflects the extent to which the cysteine residue of interest is exposed. NEM titration was conducted for all variants to determine the minimum NEM concentration required to prevent formation of the mPEG5K-labeled species. A higher concentration of NEM to meet this criterion indicates lower accessibility of the cysteine of interest.

As shown in Figure 7*C*, under basal condition in which cells were maintained in standard culture medium containing approximately 5 μM zinc, NEM concentrations in the range of 100 to 200 μM were required to prevent mPEG5K labeling. The lower NEM concentrations required for the cysteines located in the middle of IL2 indicate that the central region of IL2 is more accessible than its two ends (Fig. 7*D*), suggesting that IL2 is largely unstructured under this condition.

Under zinc depletion conditions, in which cells were treated with TPEN (a membrane-permeable, high-affinity Zn^2+^ chelator) and Chelex-treated culture medium, significantly higher NEM concentrations were required to completely block mPEG5K labeling for all tested single-cysteine variants compared with basal conditions (Fig. 7*D*). These results strongly indicate a substantial conformational change in IL2 that reduces the accessibility of the introduced cysteines.

To assess the effects of zinc repletion, two variants, I463C and N471C, were selected because the substituted residues flank the ^465^LxL^467^ motif in mouse ZIP4 (Fig. 7*C*). Cellular zinc levels were elevated by incubating cells with 10 μM ZnCl_2_ and 10 μM pyrithione, a zinc ionophore (51), followed by cysteine accessibility assays. Consistent with the trend observed above, zinc supplementation reduced the NEM concentrations required to block mPEG5k labeling for both variants when compared to basal conditions (Fig. 7*D*), indicating that zinc repletion increases the accessibility of the introduced cysteines.

Taken together, these results reveal a positive correlation between cellular zinc levels and cysteine accessibility within the IL2 loop (Fig. 7*D*). This correlation cannot be attributed to a direct effect of zinc on thiol chemistry, as zinc coordination typically reduces, rather than enhances, thiol reactivity (*i.e.* nucleophilicity in this case) (52). Instead, the observed zinc-dependent changes in cysteine reactivity reflect a conformational rearrangement of ZIP4 in which zinc binding increases the accessibility of the IL2 loop. These findings provide a structural basis for zinc-regulated engagement of the LxL motif with the cytoplasmic AP2.

## Discussion

Substrate-induced endocytosis of a transceptor represents a rapid posttranslational regulatory mechanism for maintaining nutrient homeostasis. How a transporter engages the endocytic machinery in a substrate-responsive manner is a central question, and various mechanisms have been proposed for different systems (5, 6, 8, 11, 12, 14, 15, 18). As a zinc transceptor, ZIP4 undergoes constitutive endocytosis in a zinc-dependent manner (30, 31), but the molecular basis of this multistep event remains to be elucidated. In this work, by integrating cell biology, biochemistry, and modeling approaches, along with the application of a custom anti-ZIP4 mAb to study endogenously expressed ZIP4 in cancer cell lines, we found that the LxL motif located in the IL2 loop of ZIP4 exhibits different accessibilities in response to cellular zinc levels and acts as a sorting signal that directly or indirectly interacts with the σ2 subunit of AP2 for clathrin-mediated endocytosis.

Several lines of evidence supported the key roles of the LxL motif and the dileucine motif-binding pocket of σ2 in ZIP4 endocytosis. First, the mutagenesis study indicated that the hydrophobicity of the two leucine residues is required for ZIP4 endocytosis, whereas the residue in between is dispensable (Fig. 1). Second, the knockdown experiments, combined with flow cytometry and confocal microscopy, indicated that endocytosis of ZIP4, either endogenously expressed in HepG2 cells or exogenously expressed in HEK293T cells, requires both clathrin and AP2. (Figs. 2 and 3). Third, AlphaFold 3 predicted the binding of the LxL motif to the dileucine motif-binding pocket of σ2, which was also supported by the results of MD simulations (Fig. 4). Fourth, the key role of the dileucine motif binding in ZIP4 endocytosis was demonstrated in the knockdown-rescue experiments using a panel of σ2 variants (Fig. 5). Lastly, the pull-down assays strongly indicated that ZIP4 and AP2 form a protein complex where the LxL motif and the dileucine motif binding of σ2 are crucial for their association (Fig. 6). These data are consistent with direct recognition of the LxL motif by the σ2 subunit in a manner analogous to canonical dileucine motif recognition. However, we cannot completely exclude the possibility that the interaction between ZIP4 and AP2 is mediated by unidentified adaptor protein(s) that bridge the two proteins. As such, future structural studies will be required to definitively establish the binding mode. Whether or not there is additional adaptor protein(s) mediating ZIP4–AP2 interaction should also be investigated using mass spectrometry. Another issue to be addressed is the sufficiency of the LxL motif for ZIP4 to recruit AP2. Although this motif is essential for ZIP4 endocytosis, our previous study showed that the LQL sequence fused to the cytoplasmic tail of CD8 did not induce internalization of the chimeric protein (31). Indeed, the interface between the LxL motif and the hydrophobic pocket in σ2 is too small to support a stable interaction as suggested in the pull-down assay. We therefore speculate that, in addition to the LxL motif, other structural elements in ZIP4, such as the cytoplasmic portions of transmembrane (TM) helices, may play complementary roles in the engagement of ZIP4 with AP2. As such, identification of additional contact points in ZIP4 and AP2 will also be an important direction for future biochemical and structural study.

**Figure 8:**
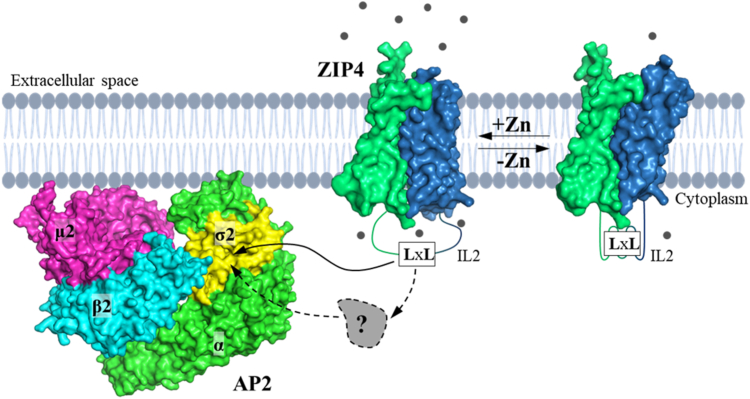
**Proposed mechanism of zinc-dependent ZIP4 endocytosis.** Cellular zinc availability regulates the conformational states of the IL2 loop, where the LxL motif is located. Under zinc depletion conditions, the LxL motif is less accessible (*right*); with increased zinc levels, the LxL motif becomes more exposed (*left*), allowing it to be recruited by AP2 (PDB: 2XA7, modeled in the membrane-bound state) through a direct binding to the dileucine motif-binding pocket in the σ2 subunit (*solid arrow*). Alternatively, unidentified cytoplasmic adaptor protein(s) (shown as a *gray* object labeled with a question mark) may mediate ZIP4-AP2 interaction (*dashed arrows*). For clarity, the ECD of ZIP4 is trimmed. The transport domain of ZIP4 is depicted as a two-domain protein with the scaffold and transport domains shown in *green* and *blue*, respectively. Alternating access is achieved by vertical sliding of the transport domain against the static scaffold domain, which triggers conformational changes of IL2. Zinc ions are depicted as *gray spheres*. Only one monomer of a ZIP4 dimer is shown for clarity. The amino acid sequences of IL2 and other intracellular loops of hZIP4 are shown in Fig. S6 for comparison. IL2, second intracellular loop; MFI, mean fluorescence intensity; ΔMFI, difference of MFI; ZIP, Zrt-/Irt-like protein; AP2, adaptor protein complex 2; ECD, extracellular domain.

The systematic cysteine accessibility assays conducted in live cells indicated that the IL2 loop undergoes substantial structural changes in response to the fluctuation of cellular zinc levels (Fig. 7). Although the IL2 loop is intrinsically disordered (28), the cysteine accessibility assay, which is sensitive to the local structure and dynamics, allowed us to conclude that the IL2 loop becomes more exposed as zinc levels increase, providing a reasonable explanation for the zinc dependence of ZIP4 endocytosis (Fig. 8). According to the proposed elevator transport mode (53, 54, 55, 56), zinc binding to the transport site triggers a global conformational change from the outward-facing state to the inward-facing state. This is achieved by a downward slide of the transport domain, a 4-TM bundle (TM1/TM4/TM5/TM6, blue domain in Fig. 8), relative to the scaffold domain (TM2/TM3/TM7/TM8, green domain in Fig. 8). Because the IL2 loop connects the two domains, the large relative movement between them is likely to induce conformational changes in IL2 that favor association of the LxL motif with AP2, thereby initiating endocytosis.

In summary, we conclude that the LxL motif in ZIP4 functions as an atypical dileucine motif to directly or indirectly associate with AP2 σ2 subunit in a zinc-responsive manner for clathrin-mediated endocytosis. Given that ZIP4 is aberrantly upregulated in cancers, elucidating its endocytosis mechanism in cancer cells will facilitate the development of antitumor therapeutics, such as antibody-drug conjugates that exploit target protein-mediated endocytosis for cellular entry. In addition, the proposed binding mode of the LxL motif to AP2 suggests that the search for sorting signals is not yet completed and that additional sorting signals may retain to be discovered in other plasma membrane cargo proteins. Indeed, several monoleucine-based motifs have been reported to be important for the sorting of basolateral proteins in polarized cells (57). Whether these monoleucine-containing motifs interact with AP complexes through the same or a similar mechanism as that proposed for the LxL motif remains an important question for future investigation.

## Experimental procedures

### Cell lines

HEK cells (HEK293T, ATCC, Cat#CRL-3216), human hepatoblastoma cell line HepG2 (ATCC, Cat#HB-8065, a generous gift from Dr Hua Xiao in Michigan State University), human pancreatic adenocarcinoma cell line AsPC-1 (ATCC, Cat#CRL-1682), and human glioblastoma multiforme cell line U251 (Sigma, Cat#09063001, a generous gift from Dr Chunqi Qian in Michigan State University) were cultured in Dulbecco’s modified Eagle medium (DMEM, Thermo Fisher Scientific, Invitrogen, Cat#11965092) supplemented with 10%(v/v) fetal bovine serum (FBS, Thermo Fisher Scientific, Invitrogen, Cat#15240062) at 5% CO_2_ and 37 °C.

### Genes and plasmids

The complementary DNA of human ZIP4 (GenBank ID: BC062625) from Mammalian Gene Collection was purchased from GE Healthcare. The DNA encoding ZIP4 were inserted into a modified pEGFP-N1 vector (Clone, Cat#6085-1) in which the downstream EGFP gene was deleted and a human influenza HA tag was inserted before the stop codon, leading to a ZIP4-HA construct. The pCMV3-based vectors for the expression of N-terminal FLAG-tagged mouse ZIP4 (GenBank ID: 72027, Cat#MG53693-NF) and the untagged human AP2S1 (σ2 subunit of AP2, GenBank ID: 1175, Cat#HG12478-UT) in mammalian cells were purchased from Sino Biological. The DNA sequence encoding the ECD of human ZIP4 was amplified and inserted into the pLW01 vector for the expression of N-terminal His_6_-tagged ZIP4-ECD in *Escherichia coli*. All the mutations were made using PfuTurbo DNA polymerase (Agilent, Cat#600250) and verified by DNA sequencing. All the plasmids were purified using Miniprep (Promega, cat#A1460) or Maxiprep (QIAGEN, Cat# 12163). All the primer sequences for mutagenesis are listed in Table S1.

### Transient transfection and RNAi

For transient expression of ZIP4 and its variants, HEK293T cells were transfected with the corresponding plasmids using Lipofectamine 2000 (Thermo Fisher Scientific, Invitrogen, Cat#11668019) and cultured for 16 h prior to the antibody uptake assay. To enhance knockdown efficiency, HepG2 cells were subjected to sequential siRNA transfection: after the initial transfection, a second round was performed 48 h later, followed by additional 48 h incubation before analysis. In the σ2 subunit knockdown-rescue experiments, siRNA-resistant σ2 or its variants were cotransfected with σ2-targeting siRNA during the second transfection, and cells were cultured for 48 h before the assay. All siRNA sequences are provided in Table S1.

### Western blot

Proteins separated by SDS-PAGE were transferred to polyvinylidene difluoride membrane (Millipore, Cat# PVH00010) and blocked with 5% (w/v) nonfat dry milk. The primary antibodies against AP2A1 (α subunit of AP2, MyBioSource, Cat# MBS416635), AP2S1 (Abcam, Cat# ab128950), clathrin heavy chain (BD Biosciences, Cat# 610499) and ZIP4 (Proteintech, Catalog# 20625-1-AP) were applied at 1:1000 dilution, and the primary antibodies against FLAG tag (Agilent, Cat# 200474) and HA tag (Thermo Fisher Scientific, Cat# 26183) were applied at 1:3000 dilution. As loading control, β-actin levels were detected using an anti-β-actin antibody at 1:2000 (Cell Signalling Technology, Cat# 4970). Primary antibodies were detected with horseradish peroxidase-conjugated secondary antibodies (Anti-rat IgG, Cell Signaling Technology, Cat# 7077S, applied at 1:6000 dilution; anti-rabbit IgG, Cell Signaling Technology, Cat# 7074S, applied at 1:2500 dilution; anti-mouse IgG, Cell Signaling Technology, Cat# 7076S, applied at 1:6000 dilution) and signals were generated by chemiluminescence (VWR, Cat# RPN2232). Images were taken using a Bio-Rad ChemiDocTM Imaging System.

### Antibody uptake assay

To study the role of the LQL segment in ZIP4 endocytosis, ZIP4 or its variants were transiently expressed in HEK293T cells. Cells were firstly washed by Dulbecco's PBS (DPBS) (Sigma-Aldrich, Cat#D8537-500 ML) once and then trypsinized by 0.25% Trypsin-EDTA (Gibco, Cat#25-200-056) under 37 °C for 2 min. The cells were then suspended in DMEM supplemented with 10% FBS, and centrifuged at 40*g* for 3 min. The cells were then resuspended in an ice-cold 500 μl DMEM supplemented with 10% FBS before Alexa Fluor 647–conjugated anti-HA antibody (Invitrogen, Cat#26183-A647; RRID: AB_2610626) was applied at 1:400 dilution. Cells were equally divided into two samples—cells in control group was maintained at 4 °C for 45 min, whereas cells in the experimental group were incubated at 37 °C for the same period of time. After incubation, cells were centrifuged at 40*g* and 4 °C for 2 min and washed three times by DPBS. Cells were then washed with an acidic buffer (DMEM+0.2%bovine serum albumin (BSA), pH 4.0 adjusted by HCl) for 45 s to strip the surface bound anti-HA antibodies, which was terminated by the addition of ten times the volume of normal DMEM. Cells were then washed with DPBS once, fixed with 4% formaldehyde for 15 min at room temperature, and washed three more times with DPBS before flow cytometry analysis.

To study endogenous ZIP4 endocytosis in HepG2 cells, cell suspension was generated by the same approach except for a longer trypsin digestion for 5 min under 37 °C. Cells were then incubated with the 1G3B2 anti-ZIP4 mAb at 4 μg/ml under 4 °C and 37 °C, respectively, for 90 min. After washing for three times by DPBS, the surface-bound antibodies were stripped with the acidic buffer, followed by fixation and wash as mentioned above. To permeabilize cells and detect the internalized anti-ZIP4 mAb, cells were incubated with Alexa Fluor 568–conjugated goat anti-mouse IgG (Thermo Fisher Scientific, Cat#A-11004) at 1:500 dilution and 0.1% Triton X-100 (americanBIO, Cat#9002-93-1) in DPBS at 4 °C for 1 h. Cells were then washed for three times and suspended in DPBS for flow cytometry analysis.

In order to exclude the dead cells in the antibody uptake assay, for both HEK293T cells and HepG2 cells, LIVE/DEAD fixable violet dead cell stain (Invitrogen, Cat#L34963) was added to cell suspensions at 1 μl/ml for an incubation with antibodies at 4 °C or 37 °C.

### Flow cytometry

Cell samples were applied to a ThermoFisher Attune CytPix flow cytometer. At least 10,000 cells were assessed for each sample. The cellular debris was excluded based on forward scatter area (FSC-A) *versus* side scatter area (SSC-A) panel (Fig. S1*B*) and single cells were selected through FSC-A *versus* forward scatter height (FSC-H) gating. To generate live/dead control group, the same amount of HEK293T or HepG2 cells were placed at either 65 °C or 37 °C for 15 min, and then mixed and stained by LIVE/DEAD stain at 4 °C for 30 min. The cells were then washed twice by DPBS before flow cytometry analysis. Based on the result of this live/dead group, the same gating was applied to all the experimental groups. MFIs of Alexa Fluor 647 (HEK293T cells) or Alexa Fluor 568 (HepG2 cells) were calculated for all live and single cells in experimental groups (incubated at 37 °C) and control groups (incubated at 4 °C). The difference of MFI (ΔMFI) between experimental groups and control groups reports antibody uptake, which serves as an indicator of ZIP4 endocytosis. All the flow cytometry data processing was performed in FlowJo 10.8.1 (https://flowjo.com/previous-versions-flowjo).

### Confocal microscopy

To visualize human ZIP4 endocytosis in HEK293T and HepG2 cells, cell suspensions in DMEM were treated with either Alexa Fluor 488–conjugated anti-HA antibody at 1:400 (HEK293T) or the 1G3B2 anti-ZIP4 mAb at 4 μg/ml (HepG2). Cell suspensions were incubated at 4 °C and 37 °C, respectively, for 45 min for HEK293T cells and 90 min for HepG2 cells. After centrifugation, cells were fixed with 4% formaldehyde and washed three times by DPBS. HepG2 cells were further treated with Alexa Fluor 568–conjugated goat anti-mouse IgG at 1: 500 dilution in DPBS plus 0.1% Triton X-100 at room temperature for 1 h HepG2 cells were then washed for 3 times by DPBS before imaging. The images for HEK293T cells and HepG2 cells taken with a 20x objective using a Nikon C2^+^ confocal laser scanning microscope are shown in Figs. 2*A* and S1*A*, respectively.

To visualize the endocytosis of N-FLAG–tagged mouse ZIP4 (WT and the CysFree-TMD variant), HEK293T cells attached on poly-D-lysine–coated coverslips and transiently expressing mouse ZIP4 were incubated with an anti-FLAG antibody (Agilent, Cat#200474) at 1: 3000 dilution in DMEM supplemented with 10% FBS under 37 °C for 45 min. Cells were then washed for three times with DPBS and fixed by 4% formaldehyde, followed by permeabilization with 01.% Triton X-100 and incubation with Alexa Fluor 488–conjugated goat anti-rat IgG (Invitrogen, Cat#A-11006) at 1: 200 dilution in DPBS under room temperature for 1 h. After three times of wash with DPBS, the coverslips were mounted on the slides with fluoroshield mounting medium–containing 4′,6-diamidino-2-phenylindole (DAPI, Abcam, Cat#ab104139). The images are shown in Fig. S5*C*. Images were taken with the same Nikon C2^+^ confocal laser scanning microscope.

Confocal microscopy was also used to compare cellular localization of internalized transferrin and 1G3B2 anti-ZIP4 mAb in HEK293T (with overexpressed hZIP4), HepG2, U251, and AsPC-1 (with endogenously expressed hZIP4) cells. Coverslips (NEST, Cat#801007) were placed in 24-well plates and coated with poly-D-lysine. Cells were seeded on the coverslips and then transfected with the siRNA oligonucleotides that target clathrin heavy chain or AP2 subunits using Lipofectamine 2000 (Thermo Fisher Scientific, Cat#11668027) or Lipofectamine RNAiMAX (Thermo Fisher Scientific, Cat#13778150). To enhance knockdown efficiency, cells were subjected to two rounds of 48-h siRNA transfection. After the second round of knockdown, cells were incubated with 4 μg/ml anti-HA antibody (for HEK293T) or 4 μg/ml 1G3B2 anti-hZIP4 mAb (for other cell lines), together with Alexa Fluor 488–conjugated human transferrin (25 μg/ml for that obtained from Thermo Fisher Scientific, Cat#T13342 or 15 μg/ml for that obtained from Jackson ImmunoResearch, Cat#009-540-050), in DMEM with 10% FBS at 37 °C for 30 to 45 min, washed extensively with DPBS to remove unbound or nonspecific bound transferrin and antibody, and fixed with 4% formaldehyde at room temperature for 10 to 15 min. Cells were subsequently permeabilized with 0.1% Triton X-100 (AmericanBIO, Cat#9002-93-1) and blocked for 1 h in DPBS containing 5% goat serum (Cell Signaling Technology, Cat#5425S), followed by incubation with Alexa Fluor 568–conjugated goat anti-mouse IgG (1:500; Thermo Fisher Scientific, Cat#A-11004) at 4 °C overnight or at room temperature for 2 h. Cells were washed by DPBS for three times before the coverslips were mounted on plain glass slides with fluoroshield mounting medium containing DAPI (Abcam, Cat#ab104139). Images were taken using a Zeiss Axio fluorescence microscope equipped with a 63× objective (for HEK293T) or the Nikon C2^+^ confocal laser scanning microscope with a 60× oil objective (for other cells) at MSU Center for Advanced Microscopy. Differential interference contrast images were also acquired on the Nikon C2^+^ confocal microscope equipped with a differential interference contrast optical module using a 40× objective.

Colocalization between transferrin (Alexa Fluor 488) and anti-ZIP4 mAb (Alexa Fluor 568) was analyzed using Manders’ colocalization coefficients in FIJI/ImageJ (https://imagej.net/software/fiji/downloads) (https://imagej.net/software/fiji/downloads) with the Coloc2 plugin. Briefly, confocal images were background-subtracted and identical regions of interest were defined for both channels within individual cells. Thresholds for each channel were determined automatically using the Costes method to minimize user bias. M1 and M2, which represent the fraction of 1 fluorophore overlapping with the other, were then calculated for each region of interest. At least 40 individual cells from two independent experiments were analyzed for evaluation.

### Pull-down assay

For pull-down assays using cell lysates as input, 20 μl protein G resin (Pierce protein G Agarose, Cat#20399) was equilibrated with the Protein G binding buffer and then incubated with 20 μg M2 anti-FLAG mAb (from 1 mg/ml stocking solution) in 200 μl protein G binding buffer at room temperature for 1 h with gentle shaking. The supernatant was discarded and the resin was washed for three times with the protein G binding buffer. Ten micrograms FLAG-tagged hZIP4, which was expressed in HEK293 Freestyle cells and purified using an M2 resin (Millipore Cat#A2220) in the ZIP4 purification buffer containing 0.0035% n-dodecyl β-D-maltoside (DDM), 50 mM Tris–HCl, pH 7.2, and 300 mM NaCl, was added to the anti-FLAG antibody-loaded protein G resin and incubated at 4 °C for 2 h with gentle shaking. The supernatant was discarded and the resin was washed for three times with the ZIP4 purification buffer. HEK293T cells that overexpressed the σ2 subunit (or the variants) were lysed in 0.5% Triton X-100 in the ZIP4 purification buffer without DDM, and the supernatant was added to the hZIP4-loaded resin for an overnight incubation at 4 °C with gentle shaking. After a 10*g* centrifugation, the supernatant was collected as flow through and the resin was washed two times with cold ZIP4 purification buffer without DDM. The proteins associated with the resin were then eluted with 0.1 M glycine buffer at pH 2.2. For the control group, all the procedure was the same except that the M2 anti-FLAG mAb was replaced by the same amount of mouse serum IgG (Sigma-Aldrich, Cat#I5381). Proteins in the elution were detected in Western blot.

For the pull-down assay under zinc depletion condition, 1 mM EDTA was added to the cell lysate that was prepared as described above. In parallel, 1.5 mM ZnCl_2_ was added to this cell lysate to make the sample for the pull-down assay under zinc repletion condition. The cell lysates under zinc depletion or repletion conditions were then applied to the ZIP4-loaded Protein G resin. All the other procedures were the same as the pull-down assay under basal condition.

### Cysteine accessibility assay

DMEM supplemented with 10% FBS was treated by Chelex 100 resin (Sigma-Aldrich, Cat#C7901-25G) to generate a Chelex-treated medium. First, HEK293T cells expressing CysFree-TMD and its variants were washed with the Chelex-treated medium and then incubated with 20 μM TPEN (Sigma-Aldrich, Cat#P4413) and 0.5% dimethyl sulfoxide (DMSO) at 37 °C for 15 min. Cells were then washed twice with the Chelex-treated medium for analysis under zinc depletion conditions. To create a zinc repletion condition, after depletion of both extracellular and intracellular zinc pools as mentioned above, 10 μM zinc chloride and 10 μM 2-mercaptopyridine N-oxide sodium salt (pyrithione, Sigma-Aldrich, Cat#H3261-1G) were added to the HEK293T cells. The DMEM supplemented with 10% FBS without treatment was used for basal condition.

The treated HEK293T cells were incubated with indicated concentrations of NEM (Thermo Fisher Scientific, Cat#23030) under 4 °C for 1 h. Cells were then washed twice to remove the excessive NEM with a buffer containing 100 mM Tris–HCl, 60 mM NaCl, 10 mM KCl, pH 7.0, and lysed with 6 M urea, 0.5% SDS, and 0.5 mM DTT (to quench residual NEM). Cell lysates were then heated for 10 min under 96 °C to completely denature proteins, followed by an incubation with 5 mM monofunctional PEG maleimide 5k (mPEG5k, Creative PEGWorks, Cat#PLS-234) to react with free thiol cysteine residue for 1 h at room temperature with gentle shaking. Samples were then mixed with SDS sample loading buffer containing 20% β mercaptoethanol and subjected to SDS-PAGE. CysFree-TMD variants were detected in Western blot using the anti-FLAG antibody.

### Zinc transport assay and inductively coupled plasma mass spectrometry

^70^Zn transport assay and inductively coupled plasma mass spectrometry (ICP-MS) analysis were conducted as described (58). In brief, 16 h posttransfection, HEK293T cells expressing mouse ZIP4 (WT and the CysFree-TMD variant) were washed with a washing buffer (10 mM Hepes, 142 mM NaCl, 5 mM KCl, 10 mM glucose, pH 7.3), followed by incubation with the Chelex-treated medium. Then, 5 μM ^70^Zn was added to cells to initiate zinc transport. After incubation at 37 °C for 30 min, cells were transferred on ice and an equal volume of the ice-cold washing buffer containing 1 mM EDTA was added to the cells to terminate metal uptake. After three washes with the ice-cold washing buffer, 70% trace nitric acid was added to cells for digestion and subsequent ICP-MS analysis. Zinc uptake by cells was expressed as the count ratio of ^70^Zn/^31^P determined by ICP-MS, and zinc transport activity was calculated by subtracting zinc uptake by the cells transfected with an empty vector from that by the cells expressing metal transporters.

### Production of anti-human ZIP4 mAbs

His_6_-tagged human ZIP4-ECD was expressed and purified in the same way as bat ZIP4-ECD (49, 50). In brief, *E. coli* strain of Origami B(DE3) pLysS (Novagen, Cat#70839) was cultured in lysogeny broth medium and protein expression was induced by 0.2 mM IPTG, followed by culturing at 16 °C overnight. ZIP4-ECD in cell lysate was purified using nickel-nitrilotriacetic acid affinity column and applied to size-exclusion chromatography after the His_6_-tag was removed by thrombin. The purified protein was delivered to Creative Biology to immunize mice. Hybridomas were screened and positive clones, including Clone 1G3B2, were identified and verified using ELISA.

Hybridoma (Clone 1G3B2) were cultured in Hybridoma-SFM medium (Thermo Fisher Scientific, Cat#12045076) and the culture supernatant was collected and clarified by centrifugation at 6000 rpm at 4 °C for 10 min, followed by filtration through a 0.22 μm membrane. The clarified supernatant was loaded onto a protein G Agarose (Thermo Fisher Scientific, Cat#20398) pre-equilibrated with protein G IgG-binding buffer (Thermo Fisher Scientific, Cat#21011). After washing with 10 column volumes of IgG binding buffer to remove nonspecifically bound proteins, bound IgG was eluted with 0.1 M glycine-HCl buffer (pH 2.9) and immediately neutralized with 1 M Tris–HCl (pH 8.0). Eluted fractions were pooled and concentrated using Amicon Ultra centrifugal filters (Millipore, 30 kDa molecular weight cutoff), and applied to buffer exchange into PBS. The final IgG concentration was determined by the absorbance at 280 nm measured using a NanoDrop spectrophotometer. Purity of 1G3B2 mAb and its specific staining of native human ZIP4 expressed in HEK293T cells are shown in Fig. S2, *A* and *B*, respectively.

### MD simulations

The predicted structural model of AP2 bound with a peptide corresponding to the segment of ^449^GVSLQLAPSE^458^ from the IL2 of ZIP4 was used as the starting model for three independent unbiased MD simulations of 500 ns each. Each system was solvated in a cubic box with dimensions of 113 × 127 × 142 Å^3^ under periodic boundary conditions. The TIP3P water model as used for solvation (59), and the systems were neutralized with 0.15 M NaCl. Energy minimization was performed in three stages for 10,000 cycles, during which the first 5000 cycles applied the steepest descent algorithm, followed by 5000 cycles of conjugate gradient minimization. The minimized systems were later equilibrated in two stages under NVT and NPT ensembles, each for 5 ns. The SHAKE algorithm was applied to constrain all bonds (60), and temperature was maintained at 300 K using the Langevin thermostat. A cutoff of 8 Å was applied for nonbonded van der Waals interactions, while Particle Mesh Ewald was used to handle long-range electrostatics. Pressure was maintained at 1 bar using the Berendsen barostat with a relaxation time of 1 ps (61). To test the reliability of the system, the predicted model containing a peptide corresponding to the segment of “^477^ESPELLNPEP^486^” in the IL2 of ZIP4 was applied to MD simulations and both leucine residues were found to stay in the hydrophobic pocket throughout the simulations, which is consistent with the notion that the pocket can accommodate two consecutive leucine residues in the canonical dileucine motif.

### Quantification and statistical analysis

We assumed a normal distribution of the samples and multiple comparisons were assessed using two-tailed Student’s *t* test. A *p*< 0.05 was considered statistically significant. Data were presented as mean ± SD.

## Data availability

Further information and requests for resources and reagents, including the plasmids and the 1G3B2 anti-ZIP4 antibody generated in this study, should be directed to Dr Jian Hu (hujian1@msu.edu) and will be fulfilled upon completion of a Materials Transfer Agreement. Any additional information required to reanalyze the data reported in this paper is available from Dr Jian Hu upon request.

## Supporting information

This article contains supporting information.

## Conflict of interest

The authors declare that they have no conflicts of interest with the contents of this article.
